# “Calling for help: I need you to listen” - A qualitative study of callers’ experience of calls to the emergency medical communication centre

**DOI:** 10.1186/s13049-023-01161-2

**Published:** 2023-12-07

**Authors:** Trine Berglie Spjeldnæs, Kristine A. Vik Nilsen, Lars Myrmel, Jan-Oddvar Sørnes, Guttorm Brattebø

**Affiliations:** 1 Nord University Business School, Universitetsalléen 11, 8026 Bodø, Norway; 2 Norwegian National Advisory Unit on Emergency Medical Communication (KoKom), Haukeland University Hospital, Bergen, Norway; 3 Department of Anaesthesia and Intensive Care, Haukeland University Hospital, 5021 Bergen, Norway; 4 Department of Clinical Medicine, University of Bergen, Bergen, Norway

**Keywords:** Emergency medicine, Emergency medical communication center, Emergency medical dispatch, Emergency medical assistance, Communication, Patients/callers, Perspectives, Trust

## Abstract

**Background:**

The Emergency Medical Communications Center (EMCC) is essential in emergencies and often represents the public’s first encounter with the healthcare system. Previous research has mainly focused on the dispatcher’s perspective. Therefore, there is a lack of insight into the callers’ perspectives, the attainment of which may contribute significantly to improving the quality of this vital public service. Most calls are now made from mobile phones, opening up novel approaches for obtaining caller feedback using tools such as short-message services (SMS). Thus, this study aims to obtain a better understanding of callers’ actual experiences and how they perceived their interaction with the EMCC.

**Methods:**

A combination of quantitative and qualitative study methods was used. An SMS survey was sent to the mobile phone numbers of everyone who had contacted 113 during the last months. This was followed by 31 semi-structured interviews with people either satisfied or dissatisfied. Thematic analysis was used to investigate the interviews.

**Results:**

We received 1680 (35%) responses to the SMS survey, sent to 4807 unique numbers. Most respondents (88%) were satisfied, evaluating their experience as 5 or 6 on a six-point scale, whereas 5% answered with 1 or 2. The interviews revealed that callers were in distress before calling 113. By actively listening and taking the caller seriously, and affirming that it was the right choice to call the emergency number, the EMCC make callers experience a feeling of help and satisfaction, regardless of whether an ambulance was dispatched to their location.

If callers did not feel taken seriously or listened to, they were less satisfied. A negative experience may lead to a higher distress threshold and an adjusted strategy before the caller makes contact 113 next time. Callers with positive experiences expressed more trust in the healthcare systems.

**Conclusions:**

For the callers, the most important was being taken seriously and listened to. Additionally, they welcomed that dispatchers express empathy and affirm that callers made the right choice to call EMCC, as this positively affects communication with callers. The 113 calls aimed to cooperate in finding a solution to the caller’s problem.

**Supplementary Information:**

The online version contains supplementary material available at 10.1186/s13049-023-01161-2.

## Background

The national toll-free phone number for emergency medical assistance in Norway is 113. The 16 emergency medical communication centers (EMCC) are organised as public services within the specialized hospital healthcare system and consist of a network of control rooms. When contacting 113, the call is received by trained and certified health personnel (registered nurses and paramedics). The police and fire rescue services have separate national emergency phone numbers (112 and 110, respectively). This system differs from many other countries, such as Denmark, Sweden, Finland, and the United Kingdom, where there is one phone number (112) for all emergencies [1]. EMCC operators play an essential role in helping callers and are the public’s first contact with the healthcare system when facing a medical emergency [2].

When handling an emergency call, the EMCC operator needs to assess the situation and decide on a solution, as a 113-call does not automatically result in ambulance dispatch. The operator gathers information from the caller, provides adequate advice and instructions, and either dispatches an ambulance or transfers the call to a local public emergency room for less urgent situations. For decision support, the dispatchers use the Norwegian Index for Medical Emergency Assistance [3].

In the context of an emergency call, the EMCC operator relies on information provided by the caller, which depends on the EMCC operator’s ability to fully comprehend and appreciate the situation. Hence, the interaction between the EMCC and the caller is vital, and understanding the dynamics of this communication is paramount [4].

It is known that being in a dramatic situation, such as a medical emergency, can profoundly affect a person’s life [5]. The nationwide project “Saving lives together” (“*Sammen redder vi liv*”) recommended further research to enhance the interaction between the caller and the EMCC [6].

Previous research focused primarily on dispatcher roles and perspectives [7, 8]. Some studies have discussed how a dispatcher’s early recognition of cardiac arrest can increase patient out-of-hospital cardiac arrest survival rates. Conversely, others have shed light on the effect of successful communication between the EMCC and the caller [9, 10]. However, to our knowledge, no previous studies have addressed this issue from the callers’ perspective. Hence, this study aimed to obtain a better understanding of callers’ actual experiences and how they perceived their interaction with the EMCC during 113 calls.

## Material and methods

### Setting

The study was conducted in Bergen, the second-largest city in Norway. The Bergen EMCC covers an area of approximately 460,000 inhabitants and handles nearly 60,000 calls annually. To get in contact with past callers to the emergency medical number, a SMS message was sent to all mobile phone numbers, from which 113 calls were made between November 2020 and February 2021 (Additional file 1). The Christmas period (10 days), and approximately five well-known individual callers, were excluded because this period differs from the rest of the year, and to protect these assumed vulnerable individuals.

The aim was to measure the general level of satisfaction, the callers’ willingness to provide feedback, and recruiting informants for the qualitative part of the study.

### Quantitative part: SMS-study

In the SMS, the callers were asked to rate their recent conversation with the EMCC on a scale from 1 to 6 (1 = “very unsatisfied” and 6 = “very satisfied”). The SMS also included a second question asking whether the respondents were willing to be contacted by the researchers for further questions. The text messages were sent only once.

### Qualitative part: interviews

The second and qualitative part of the study consisted of semi-structured interviews with the informants recruited in the first part. As we had limited previous data base our interviews on, and the wish for fully grasping the caller’s experience, we wanted the informants speak as freely as possible, but at the same time ensure some structure. Therefore, we based the interviews on a semi-structured interview guide (Additional file 2). Follow-up questions were based on the informants’ answers, inspired by the systematic methodology of thematic analysis [11]. All informants were asked the same main questions.

To test the interview guide, we conducted expert interviews with a former dispatcher with more than 20 years of experience. This also helped providing additional follow-up questions in the interview guide. The expert was specifically asked what he would have wanted to know about the caller’s experience and what kind of information would be helpful or provide insight for a dispatcher doing his job.

We then conducted 31 semi-structured interviews with 16 satisfied callers (ratings 6–4), and 15 dissatisfied callers (rating 1–3). We strived for a balanced numbers for the interviews. However, the selection did not reflect the distribution of responses, as most of the respondents were satisfied. Therefore, we chose to contact all most unsatisfied respondents (ratings 1 and 2), but could not contact the same proportion of all the most satisfied respondents (ratings 5 and 6).

Two researchers (TS and KN) conducted all interviews with the informants in March and April of 2021, either by phone (81%) or via video conferencing (19%) due to Covid-19 restrictions.

### Data analysis

Interview data collection, including notes, transcription, first-hand analysis, coding, sorting, and analysis, are important steps to ensure that data are properly processed [12]. The interviews were audio recorded, and later transcribed manually. Immediately following each interview, the researchers conducted a first-hand analysis, comparing their first impressions and interpretations. This first-hand analysis was particularly useful ensuring that the essence of each interview was identified. These sessions were recorded and helpful later in the process, permitting go back and see whether the analysis was consistent or had changed from our first impression.

This revealed a pattern emerging from the very beginning, identifying clear key words for coding, and also we quickly got saturated data.

Some codes were suitable for most informants, while some unexpected codes were emerging in several interviews. Then, the interviews were transcribed and analysed in more depth. Initially we identified 83 nodes, with 1 to 110 citations per node.

Thereafter, analysis of each code followed, merging them into relevant subgroups and main themes, as they were understood to belong under the same umbrella. The main theme categories emerged naturally, and made it possible to analyse several codes that turned into being associated. Eventually, this resulted in seven main categories as presented in Table 2.

## Results

**Fig. 1:**
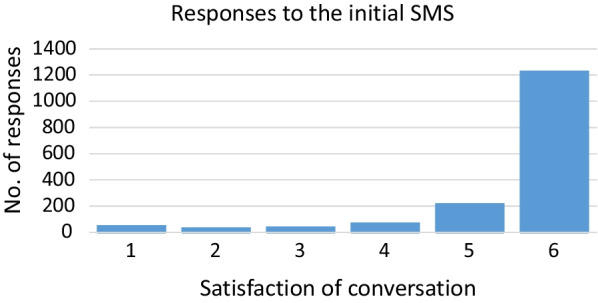
Distribution of the 1639 received responses (of 4807) SMS on the experience of the conversation with the emergency communication centre EMCC). Ratings between 1 (not satisfied), and 6 (very satisfied)

A total of 4807 SMS messages were sent to recent callers, of which 1680 (35%) responded. The vast majority (88%, rating 5 or 6) were very satisfied with the 113-call (Fig. 1).

Based on the solid responses to the SMS survey, it was evident that people needed or wanted to provide feedback on their experiences with the 113 services. The specific numbers were also the first measure of the callers’ general level of satisfaction prior to further in-depth research into this concept.

A total of 823 (49%) respondents volunteered for the interviews, making informant recruitment accessible. Of these, 50 callers were randomly, and equally selected for all values (1 to 6). We were unable to perform 19 interviews because of either unavailability or lack of answers when calling the selected numbers. The 31 callers interviewed (14 (45%) men and 17 (55%) women) had surprisingly miscellaneous background. Seven patients had a relevant medical history. Some called 113 for the first time, whereas 21 (68%) had previous experience (Table 1).

**Table 1:** Distribution of ratings by those interviewed

Rating	No. of conducted interviews	Proportion (%)
6 (very satsified)	8	26
5	4	13
4	4	13
3	5	16
2	7	22
1 (very unsatsfied)	3	10
Total	31	100

By interviewing the 31 informants, including both satisfied and dissatisfied individuals, several factors were identified. Table 2 summarizes the main results from the interview. In addition, we found that some topics were unexpectedly mentioned as important to callers. In Additional file 3 more quotes are given with their corresponding codes and main themes.

**Table 2:** Main findings from the interviews with main themes and comments

Main themes	Detailed comments and findings
High threshold for calling 113	* Calling EMCC was not an easy choice * Considered other options first * Wanted to manage on their own * Did not want to disturb, or take someone else’s place * Fear of being perceived as hysterical * Influenced by bad experiences in the past
Callers’ expectations	* Advice and urgent help from a professional * An immediate ambulance dispatch was often expected * Expectation that the EMD has access to their medical records * Considered the health care system as one unit, with good internal communication * Fear of not getting help
Context	* Callers often had several, or too many, tasks to handle * Callers were emotionally affected, felt responsible, and reluctant to complain or quit * Considerable respect for EMCC’s authority, rarely questioning decisions * Too many questions can be draining, especially when the callers themselves were the patient * Some felt alone and unsupported in the situation
Positive experiences with the EMCC operator	* Being listened to and taken seriously * Establishing an alliance with the caller * Received proper information * Explicitly informed that help was on the way * Good communication
Negative experiences with the EMCC operator	* Perceived the operator as uninterested, oblivious, unprofessional, ignorant, or arrogant * Reluctant to dispatch an ambulance * Making the caller feel stupid
Consequences beyond the present incident	* Lack of trust in the system * Blaming themselves for delayed help * Plan to change strategy for future calls * Learning medical “trigger words” * Higher threshold for calling 113 in the future
Wish for providing feedback	* The callers expressed an explicit wish for giving feedback to avoid future mistakes

Callers expressed a high threshold for calling the EMCC. None of the informants stated that calling 113 was their first choice; many other options were considered first. Several informants stated that they wanted to stay in the comfort of their own homes rather than in a hospital or emergency room. They preferred to take care of themselves, and most importantly, they did not want to become sick. Several participants also expressed concerns about being anticipated as hysterical when calling an EMCC.

Participant 24 (rating 4) described the fear of being perceived as a hysterical parent. Participant 13 (rated 2) said that although his doctor advised him immediately to call 113 when needed, he wanted to wait as long as possible before calling. Several informants said that they needed to be certain before calling for help, as they did not want to unnecessarily disturb the EMCC or exploit public resources. They did not want to place a financial burden on the healthcare system or their families and communities. Participant 28 (rated 3) described calling 113 as a difficult choice, as it could have consequences for other people in more urgent need, meaning that if they were to be helped, someone else who possibly needs an ambulance more than they would not receive a timely dispatch.

Hence, the choice of calling 113 seemed to be difficult. The informants said they would only make the call if they had no other options. Participant 3 (rated 6) described calling 113 as their *‘biggest cry for help.’* Several participants explained that bad experiences while calling the EMCC increased the threshold for future calls.

Nearly all informants with positive experiences stated that the EMCC reassured them that they had made the right decision when calling for help, even when an ambulance dispatch was not the final solution. This is particularly important if the caller has had previous negative experiences.

Even medically trained participants expressed hesitation in challenging the operator’s decisions. For example, informant 5, a nurse (rated 1), perceived that they could not ask for an ambulance. The result was a higher threshold because they dreaded calling back or did not expect to obtain sufficient help. Another instance was a call made by Informant 15 (rated 2), who wondered, after several rejections, whether it was worth calling 113.

### The caller’s expectations

All participants were expected to receive help soon after the call. Most informants had the impression that calling 113 equaled getting an ambulance and/or being admitted to the hospital. Only a few callers, most of whom had a medical background, called 113 for advice.

Several informants expected the dispatcher to know or even be able to see the location from which they were calling and that the EMCC had full access to their previous medical records. Most callers did not distinguish between the various organizational units within the healthcare system, as they considered their uniform.

Considering the callers’ high threshold for calling 113 and their deep wish to take care of themselves, many described a feeling of relief when the operator said that there was no need for an ambulance. The callers also understood that the EMCC had to prioritize resources, especially when their situation was not urgent.

### The context and acknowledging the caller’s perspective

Several informants performed well beyond expectations during their conversations with the dispatcher. Participant 26 (rated 3) described an incident in which he was talking to the EMCC while providing CPR, using a defibrillator, and simultaneously assisting the air ambulance in landing safely.

Participant 27 (rated 3) sat with the patient, a stranger that she had found on the street for almost an hour, even after he had threatened and waved her with a knife. He had collapsed on his way ‘to kill someone’ as he explicitly explained. The informant never considered leaving him because he needed help, and she did not want him to be a danger to anyone else, staying put until the police and paramedics arrived.

Participant 5 (rated 1), a registered nurse, described feeling pressured to drive her severely ill husband to the emergency room. She was certain that the patient was about to lose consciousness as his condition worsened. She was alone, driving through the city center during rush hour and combining the roles of driver and nurse. When asked if she ever considered stopping the car or calling 113 again, she replied, ‘*How could I? I had already talked to them, and they had made their decision clear. Furthermore, it was not possible as I was both driving and taking care of my husband.’* Participant 11 (rated 2) also felt pressured to drive her severely ill husband to the emergency room. In retrospect, she described this as a bad idea, as she was emotionally imbalanced, scared, and driving too fast, *‘I drove him myself, but I shouldn’t have [done so] because I was so scared, and drove so fast…*’.

In addition, the informants expressed great respect for the authority of the EMCCs. Participant 21 (rated 6) followed the EMCC operator’s advice to take a drug he knew could be potentially dangerous to him. He did not question this advice or inform the operator of his condition because he trusted his expertise. This information was an accidental finding because the informant was very satisfied with the call.

In addition to the workload of having someone in immediate distress (sometimes themselves), some callers felt stressed by EMCC operators’ numerous questions. Participant 31 (rated 3) said he became frustrated with answering several questions when he wanted to comfort the patient. Other informants, being alone at the time of the call, as it was for their sake, described finding the situation challenging and that, at the moment, even answering simple questions was a strain. Participant 21 (rated 6) said, ‘*All those questions when you’re very ill shouldn’t be necessary. (…) It’s not so easy when you’re feeling that ill*.

Some patients were less available for excessive questioning than others. They may have been in the middle of an incredibly stressful situation or calling because of an emergency concerning themselves. What is even worse was that some questions were perceived as irrelevant. Understandably, the EMCC operators wanted to talk directly to the patients; however, several informants highlighted the importance of the dispatcher listening to them as the person’s next of kin. They felt pressured to hand the phone over to patients who were not in a state of taking care of or explaining themselves to them.

The informants also described the importance of explaining why the questions were asked, especially those that might have been perceived as irrelevant or unnecessary. Participants with negative experiences often described feelings of not being listened to. For instance, a dispatcher followed a prescribed list of questions rather than listening to or asking more relevant questions. Participant 31 (rated 3), ‘*When I asked, and requested a confirmation, that the ambulance was on its way, she said that it was not. Then she asked some questions that were, to me, meaningless*.’

### Positive and negative experiences with the EMCC operator

A majority of the informants expressed that the feeling of being taken seriously and listened to by the operator was the main reason for their satisfaction with the call; an operator who genuinely listened to their story made them feel supported and taken care of. Several participants answered the EMCC follow-up questions to confirm that the incident had been taken seriously.

Participants with positive experiences described feelings of cooperation and alliance with the dispatcher. For example, holding the line until an ambulance arrives, offering to transfer the call directly to the doctor´s office, or having the doctor call the patient back are all perceived as positive elements of the 113 call. It is also important to give callers the feeling that they can change their minds and call back at any time if, for example, they regret agreeing to drive the patient to the emergency room instead of getting an ambulance.

Several informants explicitly confirmed the importance of the EMCC, confirming that it was the correct decision to call 113.

Participant 18 (rated 6) described the operator as a ‘*very nice lady,’ ‘She did great and asked all the right questions. I called, and we agreed on what I should do. So that was not a problem.’* She explained that the operator seemed to understand the situation and that the two of them, the operator and caller, cooperated to find a solution. When asked whether she felt she had the possibility to choose and decide, she answered *‘Yes, absolutely. She told me that if I wanted an ambulance, she would send it right away. It was my choice to be transported by my husband.’* The respondent further described that she genuinely felt that she could change her mind if she wanted to, as the operator had asked whether she was acceptable with that solution and that she had to feel safe about it. She described the operator’s calmness as a key factor in the success of this call.

We found that callers accept many solutions if they obtain proper explanations and information. When asked whether there was something, in particular, the informant remembered from the call and how the operator was interpreted, informant 20 (rated 5) answered that the operator was *‘pretty professional and comfortable.’* When asked about the meaning of ‘professional,’ the informant meant that ‘*She listened to what I said, took it seriously, acted upon it, and asked questions that were, in my opinion*, *relevant.’*

In contrast, some callers did not think they had taken this seriously. Participant 13 (rated 2) explained, ‘*It seemed like I was seen as someone who was just joking.’* Furthermore, she described the dreadful feeling of the operator not believing in her and that she felt she had to argue for the help she needed.

Some informants experienced delayed assistance because of prejudice. For example, informant 27 (rated 3) sat with the patient for almost an hour, even after he had almost stabbed her with a knife, and did not consider leaving him. This also demonstrates the callers’ strong feelings of responsibility. Given this and being often emotionally affected, callers will do nearly anything that the EMCC would ask them to do. Several informants described the EMCC as the authority that they were reluctant to question. It is important to note that operators and their words have a significant impact on callers.

A common scenario in which prejudice interrupted communication was calling due to intoxication. Nearly all of the informants who called for a specific emergency felt that they were not taken seriously. Participant 22 (rated 1) was explicitly told by the EMCC that they did not believe in her because of several recent non-serious calls from other young people. Informant 10 (rated 2), when he was calling due to non-alcohol related injuries, felt judged by the fact that the incident happened on a Saturday evening; ‘*I felt that the attitude was “It is Saturday evening, and falling down some stairs…,” so there must be alcohol involved. I felt that they didn’t completely believe me.’*

The informants who were dissatisfied with the call described the operator as uninterested, passive, oblivious, ignorant, arrogant, and even unprofessional or ‘tired of their job,’ as if the caller had disturbed the dispatcher by calling 113. Participant 16 (rated 2) said, *‘I felt like she was sitting there, rolling her eyes.*

A considerable number of informants said that they felt belittled or sad after the conversation with the EMCC, describing feeling rejected even though they seemed reluctant to get an ambulance. In some cases, after having to argue for help, dissatisfied informants feared not getting help if they called for another time.

### Consequences beyond the actual situation

Several informants stated that they blamed themselves for delayed or unfavorable medical assistance. They described that they might have been unclear about their communication or even made a bad impression. Participant 24 (rated 4) said, *‘I don’t know if I was unclear in my communication. I could have been sloppy and tired and not knowing exactly how to articulate myself, and that might have caused an inaccurate evaluation at the other end. But that shouldn’t be decisive for the outcome.’* The informants would replay the conversation in their minds, trying to find mistakes that they had made that caused medical assistance to be less optimal or delayed. Several participants expressed frustration and wondered what they needed to say the next time to get help.

Participant 31 (rated 3) said that he had decided if he were to call in the future. He would ask for an ambulance and then hang up, avoiding the risk of any delay if he was forced to answer many irrelevant questions. He actively planned for this alternative strategy, hoping to get more efficient help next time. Participant 24 (rated 4) described having intentionally *“…learned medical terminology from his medical doctor sister to get the EMCC operator’s proper attention in the call.”* and participant 15 (rated 2) stated that after several rejections, she wondered whether ‘*…it was even worth calling 113 at all.*”

### The callers want to give feedback to the EMCC

All informants expressed that they would gladly receive an SMS requesting feedback after a 113 call, as they were used to receiving similar SMS questionnaires after having contacted nearly all the other services. Several informants wanted to complain about their insufficient EMCC experience, but had no idea where to start. Therefore, they gave up on that thought, and several informants let it go because nobody died because of this phenomenon in the experiences described by the informants.

### Additional unanticipated findings

#### Pandemic related issues

Several informants experienced delayed help due to COVID-19-related questions at the beginning of the conversation. For Informant 31 (rated 3), these questions were asked before more important questions concerning the patient’s vital signs. Informant 11 (rated 2) perceived being refused an ambulance due to the fear of COVID-19, similar to informant 30 (rated 4), who got the impression that *“If the patient had COVID-19 symptoms, she would not get an ambulance.”* An interesting finding was that neither of the informants expressed any concern regarding fearing contamination when having to meet medical personnel and environment. They experienced life-threatening situations, and desperately requested help. Their dissatisfaction was with the delayed help due to questions about Covid-19.

#### Paramedic’s behavior

Participant 10 (rated 2) was met by degrading comments from the paramedics, wanting to determine whether they were influenced by the unsympathetic EMCC operator. Participants 16 (rated 2) and 4 (rated 6) had heard paramedics explicitly say that there was no need for an ambulance during these incidents. In the case of Informant 16, this unpleasant comment from the professional paramedic became the last memory the patient had in her home before passing away a few days later.

#### Inter-agency coordination

Informants 17 (rated 2) and 26 (rated 3) both called 113 because of incidents that required the involvement of both the police and fire and rescue departments. After informing the EMCC about the situation, both perceived the conversation as unstructured and chaotic, especially considering that they had to repeat all the information when other emergency operators joined.

#### Video calls

Video has quite recently been introduced in the emergency medical services in Norway [13]. Participants 14 (rated 5) and 22 (rated 1) accepted the use of video options for the EMCC to better understand their situations. In these two incidents, the test project had a crucial impact, as the participants felt understood and believed in their despair. *‘But then they asked me to accept a video conference so I could film him and show them that this, in fact, was true. And how did it work? Well, they saw it, then said that they would come, and then came for him.’* (Participant 22).

## Discussion

The SMS survey results showed that most callers were very satisfied with their conversations with the 113 operators. In addition, based solely on responses to the SMS survey, it is clear that people want to provide feedback on their experiences with this part of the healthcare service.

The interviews revealed surprisingly clear and consistent findings that were concordant in both the satisfied and dissatisfied contexts. First, the EMCC operator is expected to be highly conscious of all the factors affecting the caller and know that their words matter profoundly. This is a study on how human beings experience life situations, and rather extreme life situations. As these often are life-changing, the experience of receiving help or not in such circumstances are profound. In this respect, the interviews yielded clear findings, as discussed above.

As evident from this study, dispatchers must remember that every individual situation has its context, even when it shares similarities with other comparable situations. This highlights the importance of obtaining a correct and thorough understanding of each unique situation as soon as possible [14–16]. By carefully choosing words and trying to achieve a meaningful communication, e.g. using open-ended compared to close-ended questions, the EMCC operators quickly can gain the necessary information [17].

Møller and colleagues analyzed several thousand emergency calls, with the result that the most frequent category of a call was “unclear problem”, in addition most calls being deemed as urgent. Especially these two factors, even more when combined, showed the need to improve the support of the operators [18]. Our study supports the need for any additional tools provided for the operator to help them in challenging unclear and urgent situations. For instance, the use of video calls proved to be highly effective.

As Roivainen et. al showed in their observational pilot study in 2020, that proper telephone triage by nurses can reduce non-urgent EMS missions by one third [2]. This implies telephone counseling, care instructions and patient guidance to other services than EMS. Meaning that a significant amount of the situations the callers express the need of help for can be met by communicating with the EMCC operator. If the operator is highly conscious of how the callers are met, this study shows that a reduction in ambulance missions will not equal dissatisfied callers. Callers and patients that do not need urgent care can be treated equally well, by others means, as long as the operator communicates in a caring way.

After all, it is much more challenging to gain sufficient information over a telephone. Salk et al. described that there were evidently poorer agreements between assessments of the same patient in person, compared to over the phone [19]. This shows that it is essential that the operators constantly are aware of this barrier in providing proper help to the caller, including for instance at times listening more to the next of kin than to the patient. Lindström and colleagues reflected on the need to increase our understanding of how ill patients communicate with professionals, especially over the phone [20]. This study found that the threshold for calling is high; many fear they are a burden or being perceived as hysterical. These factors may cause that important pieces of information either are held back or not communicated at all. The callers might be overwhelmed by the situation, either as they are emotionally affected and/or overwhelmed by the tasks they are handling. Many also have a tremendous respect for the EMCC authority, affecting the communication.

It has also been found that barriers and opportunities related to the EMSS operators or the callers are the main factors influencing the assessments of the calls [20]. In addition, with both the barriers and opportunities, communication from the professional side was among the factors. A barrier was the structure of the call, and not focusing on additional information in the call, such as the caller’s breath. Then the main issue could be lost in the caller’s description of other, less severe symptoms. Another identified barrier was lack of structure in the call. Proper communication is always the professional’s responsibility. Then important information could be lost, due to several reasons. An opportunity was the operator’s use of different communication strategies such as closed loop communication. Meaning the operator repeating and/or concluding the information given by the caller in the same form, and the caller confirming or correcting the conclusions. This correlates with this study, finding that listening with genuine care and interest is essential. If the EMCC operator’s focus on a mindful interaction with the callers, taking them seriously, explaining properly, preferably establishing an alliance, it would be easier to ask correct follow-up questions to find the best conclusions for each individual caller. Holmström and colleagues emphasize the same factors [21]. They also highlight the challenges from the professional’s side in an emergency call. Of the identified themes of challenges generated in their study, specifically calls from third parties, and calls about unclear situations, were evidently challenging for several informants. This showed that calls operators find challenging, often are felt the same way for the actual callers. Lack of visual cues and knowledge about the patient, time pressure, and the fear of making mistakes, all are factors aggravating the operators’ situational awareness and ability for optimal performance.

## Limitations

The response rate was 35%, but relative to other SMS surveys, it is rather high. Other callers may have expressed opinions that were not reflected in the 31 interviews. The data from the satisfied callers proved to be surprisingly consistent, but due to available resources, it was impossible to contact all informants who agreed to participate in the interview. To obtain a balanced view, we intended to interview 50 persons, but ended up interviewing 31 respondents. We do not have any insight into reasons why other callers did not respond to the SMS, and do therefore not know all reasons behind the lack of feedback. Language barriers could be one such reason, but it was impossible to further research this.

We do not know anything about the callers who did not respond to the SMS. Anyway, the information received during these interviews was consistent, resulting in saturation. Another limitation is that the number of unsatisfied respondents was low, and the selection did not reflect the balance of responses in the first part of the study. Two researchers conducted all the interviews to reduce the risk of personal preferences influencing the results.

A challenging identified limitation is that the 31 informants were, based on what was possible to find out over the phone or the screen, quite a homogeneous group: native Norwegian men and women. One might wonder whether for instance non-native Norwegian did not answer, due to language barriers. This was not something we could research this time, but is definitely an interesting aspect. On the contrary, the 31 informants were a very diverse group when it came to gender and age. Other identification aspects were impossible to discover. This probably led to an unintended first-hand selection even before we started calling the informants.

A factor that would be interesting to look further into is the fact that EMCC operators are individuals of personal variabilities as it is likely that this may affect a caller’s experience. This study shows the importance of the human factor in these interactions, meaning that an operator’s personality is of significance, not only the medical expertise.

## Conclusion

Most callers seemed to be satisfied with the services provided from the EMCC. This study’s findings conclude that the result of the conversations, the most applicable for a 113 call being ‘getting an ambulance or not’ is not the main criterion determining whether a caller is satisfied with the EMCC service. What matters is how the dispatcher treats them. They want appropriate help. They might initially call for, and expect an ambulance, but our study demonstrates that they will accept and be content with a given solution as long as they feel listened to and taken seriously by a genuinely caring medical professional.

In every situation, the caller knows his or her situation the best. If a professional strives to establish an alliance with a caller, it will be beneficial for the patient and better solve the situation. We found that addressing the caller’s expectations by confirming that it was the right decision to call, seemed efficient in establishing an alliance between the caller and dispatcher.

Therefore, we recommend that the EMCC personnel must focus on communication as their most important tool, always explicitly assuring the caller that it was the correct decision to call 113 and establish an easily accessible method for providing feedback. Implementing SMS surveys as used in this study may be a standard procedure for exploring callers’ evaluation of EMCC services. This will be easily accessible to the caller and may function as real-time monitoring of user satisfaction.

### Supplementary Information

**Additional file 1**. SMS-text sent to those mobile numbers that had contacted emergency number 113

**Additional file 2**. Interview guide.

**Additional file 3**. Informant quotes from the interviews, with corresponding codes and main themes

## Data Availability

The dataset from this study is available from the corresponding author upon reasonable request.

## Abbreviations

EMCC: Emergency medical communication centre
EMD: Emergency medical dispatcher
