# Supplementary material for: “Calling for help: I need you to listen” - A qualitative study of callers’ experience of calls to the emergency medical communication centre
Source: Scand J Trauma Resusc Emerg Med. 2023 Dec 7;31:94. doi: 10.1186/s13049-023-01161-2 (PMC10704617; doi:10.1186/s13049-023-01161-2)
Supplement: Supplementary file 1 — Additional file 1. SMS-text sent to those mobile numbers that had contacted emergency number 113 [file 13049_2023_1161_MOESM1_ESM.docx]

**Supplement file 1**

**SMS-text sent to those mobile numbers that had contacted emergency number 113**

Hello!

You recently called 113. We want to provide best possible help, and would therefore kindly ask for your feedback on how you experienced the conversation.

How satisfied were you with the way you were taken care of by 113?

Please answer by sending a rating between 1 (not satisfied), and 6 (very satisfied).

Would you allow us potentially to contact you for further questions regarding this emergency call?

If you do, answer YES in the same SMS.

Kind regards,
