# Supplementary material for: “Calling for help: I need you to listen” - A qualitative study of callers’ experience of calls to the emergency medical communication centre
Source: Scand J Trauma Resusc Emerg Med. 2023 Dec 7;31:94. doi: 10.1186/s13049-023-01161-2 (PMC10704617; doi:10.1186/s13049-023-01161-2)
Supplement: Supplementary file 2 — Additional file 2. Interview guide. [file 13049_2023_1161_MOESM2_ESM.docx]

**Supplement file 2**

**Interview guide**

| **Questions** | | **Follow-up questions** |
| --- | --- | --- |
| Q 1 | What were your expectations when you called 113? | Who do you imagine you are talking to?  What did you expect was going to happen? |
| Q 2 | Did you felt taken care of by the  dispatcher? | To what extent did you experience the dispatcher as empathetic, pleasant, professional, dismissive, uninterested, unfriendly? |
| Q 3 | Did you feel that the dispatcher  understood you, and did he/she  Understand the situation you called for? | Did you have to explain yourself?  Did the dispatcher have any follow-up questions? |
| Q 4 | Did you get the help you wanted  (an ambulance)? | If not, how did you feel about that? (Was the caller convinced by the dispatcher that there wasn’t a need for an ambulance? Or did you feel rejected?) |
| Q 5 | Did the conversation start off with you having confidence in 113? And was this trust in any way affected by the conversation? | Have you previously called 113?  Do you have any first aid/emergency medicine training? |
| Q 6 | If the experience was good; What led to the experience being good? | Something that was said, or the way it was said?  To what extent did you experience the dispatcher as empathetic, pleasant, professional, interested, listening? |
| Q 7 | If the experience was bad; what led to the experience being bad? | Something that was said, or the way it was said?  Did you feel frustrated during the conversation?  To what extent did you experience the operator as dismissive, uninterested, unfriendly? |
| Q 8 | How have you been after the incident /  conversation? | Have you subsequently experienced challenges that you think may have a direct connection with the experience?  If so, which ones?  Have you needed help processing these?  If so, have you sought help?  If so, did it work? |
| Q 9 | What feeling were you left with  regarding your own efforts during the  event? | If bad, how could this have been handled?  Do you feel that it could have helped if the dispatcher had given you feedback and acknowledged your effort? |

| Q 10 | How was the first night and day  after the experience?  Or: Have you thought about the  incident afterwards? How? Please elaborate. | Did you experience any reactions after the  incident? Did you sleep well? Appetite?  Did you get any information about common reactions after such incidents? If yes to the first question and no to the last - do you think this would have helped you?  How was the time after the first evening and night? |
| --- | --- | --- |
| Q 11 | Would you have wished there had been some kind of follow-up after the experience? | If so, what did you miss? What would have been desirable for you?  Would a debrief with the professional  emergency services on site have been desirable? |
| Q 12 | What is your take about the dispatchers giving you information about common after-reactions, and specific information about where you could seek help in case of discomfort after the experience? | If so, what information would have been of importance to you? |
| Q 13 | With the experience more at a distance - what do you think / feel about it today? | Do you often think about the event itself?  Do you think about the conversation you had with 113? |
| Q 14 | In hindsight, is there anything you would wish that the dispatcher had asked or said? |  |
| Q 15 | Is there anything you would like to add? |  |
