# Supplementary material for: “Calling for help: I need you to listen” - A qualitative study of callers’ experience of calls to the emergency medical communication centre
Source: Scand J Trauma Resusc Emerg Med. 2023 Dec 7;31:94. doi: 10.1186/s13049-023-01161-2 (PMC10704617; doi:10.1186/s13049-023-01161-2)
Supplement: Supplementary file 3 — Additional file 3. Informant quotes from the interviews, with corresponding codes and main themes [file 13049_2023_1161_MOESM3_ESM.docx]

**Supplement file 3**

**Informant quotes from the interviews, with corresponding codes and main themes**

- Codes: caller’s perspective; being taken seriously; COVID-19; caller-patient workload **🡪 Main themes: Context, Negative experiences**
  Informant 5, a nurse (rated 1): perceived that they could not ask for an ambulance: “The operator did not want to listen. I was only told to call the emergency room. We had been pressured that we would be able to make it, that I would be able to drive them. My spouse wanted to, and I wanted to. It wasn’t like we didn’t want to drive. I felt pressured to be able to make it.”
- Codes: caller’s perspective; caller-patient workload **🡪 Main theme: Context**
  Participant 31 (rated 3): said he became frustrated with answering several questions when he wanted to comfort the patient: *‘I just wish she could have quit all those additional questions, so I could be present in the situation. And I felt I could have helped the man and comforted him because he was a bit anguished and sad, but instead, I was talking to her on the phone.’*
- Codes: caller’s perspective; context; who are you talking to **🡪 Main themes: Context, Consequences beyond the present incident**
  Informant 24 (rated 4): *‘Sometimes they want to talk to her, and I get the point. Then I always try to explain that she is a medical doctor, so if you ask her what’s wrong, she will say that nothing is wrong. She always understates her symptoms. She always complains to me, but as soon as the EMCC is on the line, she says that she is fine.’*
- Codes: caller’s perspective; being taken seriously; context; situational awareness; who you are talking to **🡪 Main themes: Negative experiences with the EMCC operator, Context, Wish for providing feedback**
  Participant 16 (rated 2): *“…described the patient as very quiet and too sick to talk on the phone. Due to this condition, she worried that the EMCC operator would not understand the severity of the situation and therefore wanted to provide this information instead. ‘It is a question of life or death, so they must receive the information they get, and as next of kin, one must be heard. One has to be taken seriously, and no good comes from being disregarded. And it is more important, instead of getting annoyed or seeing us as disturbing.’*
- Codes: caller’s perspective; well performing operator; showing interest; care **🡪 Main themes: Positive experiences with the EMCC operator, Context**
  Participant 13 (rated 2): *“…emphasized questions such as, ‘Is the door open for when the paramedics arrive?’ or ‘Are you able to open the door?’ had previously given him a strong feeling of consolation and that the operator understood the situation.*
- Codes: caller’s perspective; being taken seriously; co-operation **🡪 Main theme: Positive experiences with the EMCC operator**
  Participant 15 (rated 2): *‘The times where a positive conversation took place are the ones in which we have good dialogue, [and] we have agreed about things together – we have made a plan.’*
- Codes: being taken seriously; co-operation; well performing operator; workload ;positive feedback **🡪 Main theme: Positive experiences with the EMCC operator**
  Participant 1 (rated 6):*‘The operator kept me very good company, and everyone helped us in cooperation; four unknown people, five if you include the EMCC operator. You were kind of a team, all of you, both the people on the site and the guy at the EMCC. Yes, we felt so’.*
- Codes: being taken seriously; dialogue and co-operation; well performing operator; acknowledging **🡪 Main theme: Positive experiences with the EMCC operator**
  Participant 24 (rated 4): *‘It was sort of that we got a confirmation that it was the right decision to call now, and that was strengthened when they [EMCC] said they understood that it was a difficult decision to make, but that we would never hesitate to call again later. Therefore, it was very comforting to know that there was someone at the other end and that it was not wrong to call. She explicitly said that it is better to call one time too many than one time too few.’*
- Codes: prejudices; substance addiction; delayed help; human worth; discrimination; unprofessionalism **🡪 Main themes: Negative experiences with the EMCC operator, Consequences beyond the present incident**Informant 27 (rated 3): *“…was asked about the seemingly unconscious patient’s description before being asked whether he was breathing. ‘Then they asked if I could describe the man, and so I did. So they asked how he looked and asked me to describe his appearance before asking whether he was breathing. I answered yes, and then they asked if this was his name. I asked him, and he confirmed, and I answered them.”*
- Codes: caller’s perspective; being taken seriously; “bad” operator; left impression; change strategy **🡪 Main themes: Negative experiences with the EMCC operator, Consequences beyond the present incident**
  Participant 13 (rated 2): *‘First and foremost, she showed little interest. She seemed so passive. It almost seemed as if she was eating at the same time as she was talking. I felt it was hopeless. It was as if I was not taken seriously at all. I was wondering, did she give up now, or is she eating, or what is she doing? It was so annoying. I wanted to hang up and call back to see if another person picked up the phone.’*
- Codes: being taken seriously; change strategy **🡪 Main theme: Consequences beyond the present incident**Participant 28 (rated 3): *‘Because you are afraid you will be taken less seriously then? Yes. Afraid that you will almost have to act in order to show how bad it is? Yes, more to that side. And I have done that in the past’.*
- Codes: caller’s perspective, authority and power balance **🡪 Main theme: Context**
  Participant 16 (rated 2): *‘So, one is a little afraid to speak up – because how will that affect the patient? Because there is something about challenging the power, and there is an imbalance in power in that situation? Very much so.’*
- Codes: caller’s perspective; how to create a “bad” caller; change strategy; wrong focus **🡪 Main themes: Context, Consequences beyond the present incident**Participant 31 (rated 3): said that he had decided if he were to call in the future. He would ask for an ambulance and then hang up, avoiding the risk of any delay if he was forced to answer many irrelevant questions. He actively planned for this alternative strategy, hoping to get more efficient help next time: *‘I thought a lot about it afterward, and there is a new tactic I might follow next time. Just call them, inform them where I am, that I need help, and that they have to come immediately. And then I would hang up the phone. I think that could have been a tactic, instead of talking to her for so long and getting all those irrelevant questions.’*
